# Social Participation and Disaster Risk Reduction Behaviors in Tsunami Prone Areas

**DOI:** 10.1371/journal.pone.0130862

**Published:** 2015-07-08

**Authors:** Nopphol Witvorapong, Raya Muttarak, Wiraporn Pothisiri

**Affiliations:** 1 Faculty of Economics, Chulalongkorn University, Bangkok, Thailand; 2 Wittgenstein Centre for Demography and Human Capital (IIASA, VID/ÖAW, WU)/Vienna Institute of Demography, Austrian Academy of Sciences, Vienna, Austria; 3 International Institute for Applied Systems Analysis, Laxenburg, Austria; 4 College of Population Studies, Chulalongkorn University, Bangkok, Thailand; Hamamatsu University School of Medicine, JAPAN

## Abstract

This paper examines the relationships between social participation and disaster risk reduction actions. A survey of 557 households in tsunami prone areas in Phang Nga, Thailand was conducted following the 2012 Indian Ocean earthquakes. We use a multivariate probit model to jointly estimate the likelihood of undertaking three responses to earthquake and tsunami hazards (namely, (1) following disaster-related news closely, (2) preparing emergency kits and/or having a family emergency plan, and (3) having an intention to migrate) and community participation. We find that those who experienced losses from the 2004 tsunami are more likely to participate in community activities and respond to earthquake hazards. Compared to men, women are more likely to prepare emergency kits and/or have an emergency plan and have a greater intention to migrate. Living in a community with a higher proportion of women with tertiary education increases the probability of engaging in community activities and carrying out disaster risk reduction measures. Individuals who participate in village-based activities are 5.2% more likely to undertake all three risk reduction actions compared to those not engaging in community activities. This implies that encouraging participation in community activities can have positive externalities in disaster mitigation.

## Introduction

Following the Indian Ocean Tsunami of December 2004, there has been an increased awareness of the potentially destructive impacts of tsunamis and other extreme natural events. Coastal communities are becoming increasingly vulnerable to natural hazards partially due to an increase in climate-induced extreme events and global environmental change as well as population growth and development in coastal areas. In order to limit the adverse impacts of natural hazards, disaster risk reduction has become a central theme of many international development agencies [1].

Minimizing disaster damages can be done on a variety of scales. At the level of the national or local government, examples of disaster mitigation measures include improving forecasting and warning systems, enhancing community resilience through promoting awareness of potential disaster risks, disseminating knowledge about disaster preparedness, and a more sensible management of environmental and natural resources [2]. These measures are essentially non-excludable public goods. At the individual level, protective measures are important, particularly when one lives in high-risk zones. Common protective measures range from storing emergency food and water supplies, preparing a household emergency plan and attending a first-aid course to purchasing insurance against natural disasters. Emergency preparedness allows households to carry out appropriate responses if/when a disaster strikes [3] and strengthens their capabilities to cope with the aftermath [4].

Nevertheless, disaster risk reduction is not a completely individual effort as it can also be fostered by social networks. Efforts to promote disaster risk reduction often emphasize the importance of community involvement. While external agencies such as governmental or non-governmental organizations may initiate disaster management and risk reduction programs, the sustainability of such activities primarily depends on partnership, participation, and ownership of local communities [5]. At the same time, community involvement in hazard mitigation also includes community empowerment in negotiating with and engaging supra-local actors such as local and central government agencies to support community-driven processes. This suggests that local resilience to natural hazards can be promoted through collective action that supports effective responses.

Recent literature has introduced social capital as a key element in disaster risk reduction. The term “social capital” is defined as a community-level as well as an individual-level attribute. Social capital, when seen as embeddedness in social networks [6] or the social structure composed of individuals and organizations, can be useful in prevention, preparation, and coping with disasters in many ways. Social networks have a diversity of functions, from sharing of expertise and resources [7] and transmission of information to supporting policies and practices that contribute to greater preparedness and effective responses [8,9]. In this sense, social capital can be deemed as a public resource that enhances the well-being of the community.

Social capital can also refer to an individual level attribute and, at the individual level, the term is sometimes used interchangeably with “social participation” [10,11]. There is a quasi-private component of social capital that can be invested in, exchanged and inherited [12]. Participation in community activities such as volunteering, religious involvement, or membership in an association is a common example of how one can invest in social capital. Social participation allows people to interact, create networks to disseminate information and provides a venue to create trust among group members [13,14]. Similar to human capital, social capital is an important determinant of human well-being as noted by Dasgupta [15] “social capital is a private good that is nonetheless pervaded by externalities, both positive and negative”. There is evidence that those who engage in social and club activities have lower risky health behaviors [11] and better self-rated health [16]. Social participation may promote disaster risk reduction behaviors in the same manner.

Indeed, it has been shown across different national contexts that social capital contributes to disaster prevention and risk reduction. For example, it was reported that residents in Charleston, North Carolina who had stronger social support were more likely to evacuate before Hurricanes Hugo and Andrew than those with weaker social support [17]. Similarly, membership in a social organization is found to increase support received following a hazard event [18,19]. On the other hand, isolated individuals are less likely to be rescued, evacuate, or receive assistance [20] and have a greater mortality risk [21]. Therefore, it can be expected that well-connected individuals should benefit from their social ties in preparation for and response to emergencies.

Regardless of the definition or the level of social capital in consideration, it is clear that social capital affects disaster preparedness [22–24]. Social networks provide channels through which a perception of risk and motivations to take preventative action can be transferred. Cohesive communities are generally more prepared for hazard events since members are more willing to collaborate on solving common problems [25]. At the individual level, those who participate regularly in social activities can benefit from an exchange of useful information and warnings, especially in times of emergency.

## Determinants of Risk Reduction Actions

With respect to other characteristics that influence disaster risk reduction behaviors, previous studies have shown that preparedness actions vary considerably with personal characteristics and circumstances. Socio-demographic characteristics including age, gender, marital status, number of children, and education are reported to be associated with disaster preparedness [26–30]. The level of preparedness is also found to increase with economic status such as income and home ownership [29,31,32].

Apart from demographic characteristics, disaster experience, as an important psychological factor, can change response activities considerably. It may alter the understanding and perception of risk and encourage that precautionary measures be undertaken. The extent to which disaster experience has an impact on self-protective behavior varies according to different components such as the number of disasters experienced [29], how recent the experience was [33], and whether losses were incurred from the disaster [34,35].

Likewise, disaster experience can also influence social capital. Social capital may be eroded following a disaster, as network members may be dislocated or lost through injury or death and network resource capacity can be overwhelmed [36,37]. However, disaster experience may renew or enhance social capital in a community during the disaster period. In “normal” times, citizenship obligations are modest; whereas in times of natural disasters, as community members share the same experience, they may feel more attached to each other. In this case a sense of belonging is generated and gains from cooperation are better realized [38]. In high risk areas, being regularly exposed to natural disasters induces communities to diffuse information concerning preventive measures and enables them to cope with risks through collective learning [24]. The experience reinforces social trust and community participation [24,39], which are useful in risk reduction.

**Fig 1:**
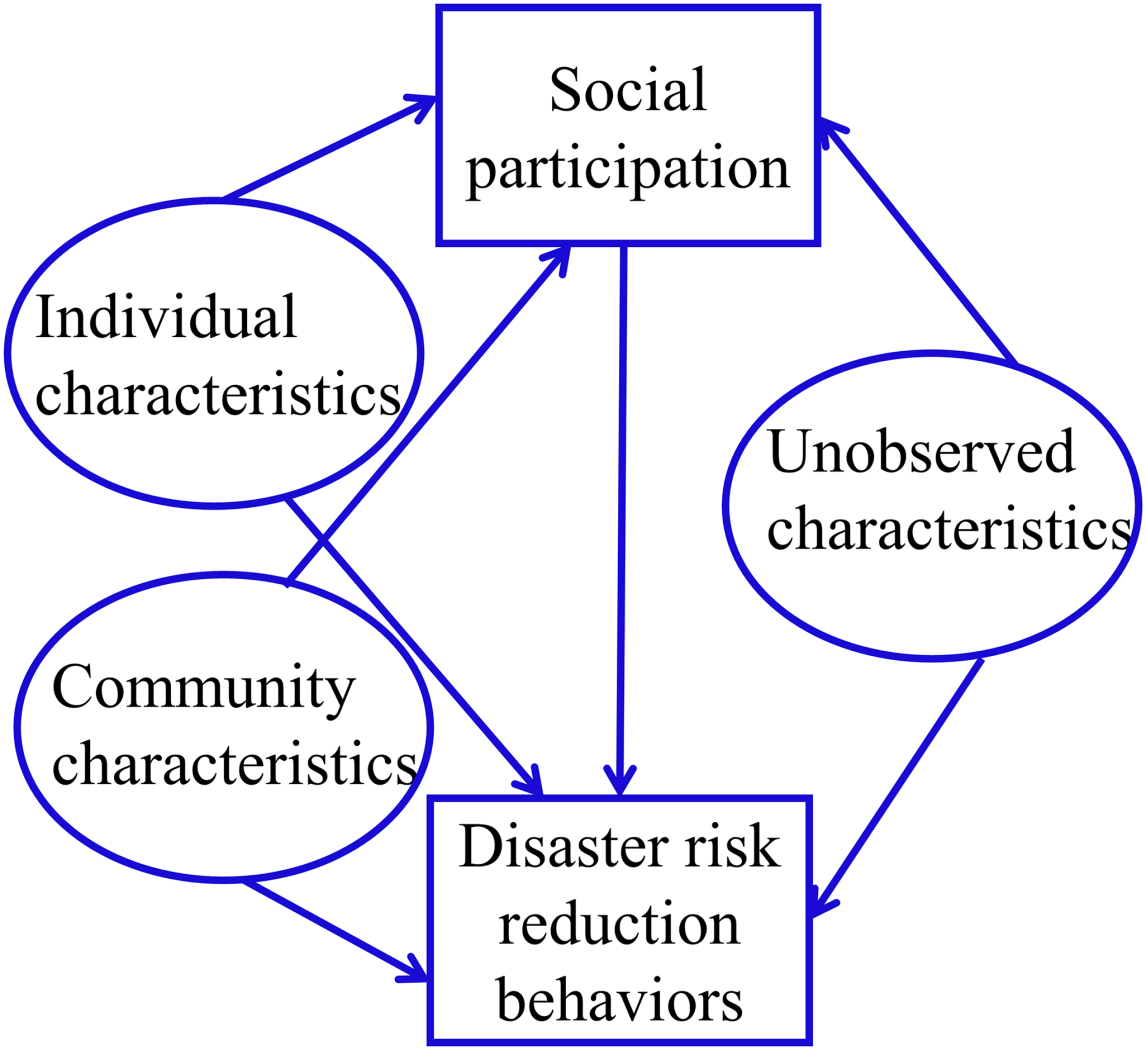
Relationships among individual and community characteristics, social participation and disaster risk reduction behaviors.

The literature clearly suggests that disaster risk reduction actions are determined by several factors, and disaster risk reduction behaviors and social capital can impact one another. Fig 1 summarizes determinants of risk reduction behaviors and social participation as well as relationships among them. The different shapes in the figure reflect the distinction between variables that are outcomes and those that are determinants in the empirical model. Risk reduction behaviors and social participation are the two outcomes of interest and they are determined by individual characteristics and community characteristics that are observable and exogenous. They are also influenced by individual characteristics that are unobservable. Examples of unobserved characteristics include risk preferences, risk perception, attitudes or beliefs, all of which cannot be captured by the data. It is hypothesized here that if the individual has unobserved characteristics that influence him/her to undertake one disaster preparedness action, those unobserved characteristics should also impact the individual’s decision to undertake other disaster preparedness actions as well as to participate in community events and vice versa. The presence of unobserved characteristics implies that, at the individual level, all outcomes of interest are jointly determined and therefore they should be estimated simultaneously.

While current empirical studies have shown that having greater individual-level social capital (e.g., perception of trust and fairness) and living in communities with higher social capital (e.g., cohesive and close knit, higher number of civic organizations) are positively associated with disaster prevention, preparedness and recovery [23,40–42], these studies commonly estimate disaster preparedness outcomes as a function of social capital (regardless of its level) and assume social capital to be exogenous. Few studies have considered the possibility that the investment in social capital and risk reduction behaviors can be jointly influenced by the same underlying characteristics.

This paper thus aims to explore determinants of and examine relationships between disaster risk reduction behaviors (measured as disaster preparedness and migration intention) and social capital (measured as social participation). It uses a survey of 557 households located along the western coastline of Southern Thailand in Phang Nga province, conducted immediately after the 11 April 2012 Indian Ocean earthquakes. Controlling for individual and community characteristics and accounting for unobserved heterogeneity, we hypothesize that disaster experience influences both social participation and undertaking of actions to reduce disaster risk, and that social participation positively influences the level of disaster preparedness.

The rest of this paper is organized as follows. The next section describes the area chosen for a case study and potential disaster risks in the region. Then, the data used for the analysis and outcome variables are discussed. Next, the multivariate probit model employed to estimate disaster risk reduction actions and social participation is explained. The results are then shown and discussed. The last section concludes the findings.

## Study Area and Context of Disaster Risks

Phang Nga province was chosen for the study because the province was the worst affected area of the six tsunami-affected provinces in Thailand in 2004. The province suffered the greatest human loss and incurred a massive economic impact due to damages to buildings and basic infrastructure [43]. Following the 2004 Indian Ocean tsunami, tsunami warning systems were installed and regular drills were introduced. The Department of the Disaster Prevention and Mitigation (DDPM) yearly organizes a full-scale tsunami warning and evacuation exercise in tsunami-risk areas in six Andaman coastal provinces. Tsunami experience together with various campaigns for disaster risk reduction were expected to have raised awareness and encouraged risk reduction actions among Phang Nga residents.

The 2012 Indian Ocean earthquake provides a unique opportunity to investigate risk reduction behaviors of households residing in tsunami-risk areas in Phang Nga. On 11 April 2012, a powerful magnitude 8.6 undersea earthquake struck 434 kilometers southwest of the Indonesian province of Banda Aceh in northern Sumatra. It was followed by another major shock (M8.2) as well as numerous aftershocks [44]. This triggered a tsunami warning for countries along the Indian Ocean rim including six provinces located along the western coastline of Southern Thailand. Although a massive tsunami did not occur because, unlike in 2004, the tectonic plates shifted horizontally rather than vertically, the event was seen as an actual test of the warning system and evacuation procedures [45,46].

The April 2012 Indian Ocean quake triggered numerous earthquakes of M4.5 and greater worldwide [47]. In particular, on 16 April 2012, an earthquake of 4.3 magnitude struck Phuket with its epicenter at Thalang district, 22 kilometers away from Phang Nga. This quake was followed by a series of more than 26 aftershocks between 16 and 22 April 2012. During that period, both the 11 April Indian Ocean quake and Phuket quakes sparked fear among locals and tourists especially in the areas previously damaged by the 2004 tsunami. Rumors were spread that Phuket could be submerged due to the quakes. Residents in the region were put on high alert for fear of a disaster similar to that of 2004.

## Data

The analysis is based on two data sources: individual- and community-level data. The data at the individual level are obtained from a survey of households located in areas at risk for tsunami conducted by the College of Population Studies, Chulalongkorn University between 17 April– 13 May 2012, immediately after the Indian Ocean earthquakes and during and just after the period of the minor earthquakes. The period of the survey was timely as risk reduction behaviors could be observed in the moment when preparedness was being tested by real events.

The process of collecting the individual-level data can be described as follows. First, the list of seven sub-districts that had been issued tsunami warnings on 11 April 2012 was obtained from the DDPM. Then, nine villages within the seven sub-districts were randomly selected as survey sites. In each village, 30% of the households were selected through systematic random sampling, where the first household was randomly selected and every third household was chosen for an interview. Households in which no member was present at the time of the first visit were not omitted but were revisited later. The survey was administered via face-to-face interviews in the Thai language by trained interview staff and local researchers. In total, 640 households were approached, of which 563 households were successfully interviewed, giving the response rate of 88%.

The survey staff first approached the head of household; in the head of household’s absence, the spouse or a household member aged 15 years or older was asked to participate. Data collection was approved by the review board of the College of Population Studies, Chulalongkorn University. Participants were explained that interviews were voluntary and that information provided would be used for research purposes only. Verbal consent was obtained before the interview; see S1 File. Participant Information Sheet. The information was anonymized and de-identified prior to analysis.

The survey questions range from basic demographic and socio-economic characteristics of respondents and their households to awareness of, response to, and preparedness for the 2012 Indian Ocean earthquakes. Questions regarding experience of the previous 2004 tsunami, social activities engaged in, and channels of information received were also included. The final sample consists of 557 households with valid responses to all questions used in the analysis.

The community-level data come from the 2010 Population and Housing Census, supplied in an aggregated form by the National Statistical Office, Thailand. The Census contains a basic demographic profile and education at the village level. Given that the Census is conducted every ten years, the 2010 Census has the narrowest time interval with the individual-level survey conducted in 2012.

### Outcome variables

There are four outcomes of interest: three disaster risk reduction actions and social participation. The three disaster risk reduction actions are: 1) following disaster-related news closely; 2) preparation of emergency kits or having a household emergency plan; and 3) migration intention. The first outcome is derived from a question in the survey, which asks: “Have you or your family members followed the news (about earthquakes and tsunami) closely after the 2012 Indian Ocean earthquake?. The second outcome is constructed from two questions: “Have you or your family members prepared emergency kits after the 2012 Indian Ocean earthquake?” and “Have you or your family members formulated an emergency plan after the Indian Ocean earthquake?”. The third outcome is derived from a question which asks: “Have you or your family members thought about moving to other areas after the 2012 Indian Ocean earthquake?”. All disaster risk reduction outcomes are binary variables; the value of 1 is assigned if the individual (or his/her family members) reported having undertaken the action and 0 otherwise.

The construction/selection of the three risk reduction measures needs to be justified. First, it is argued here that following disaster-related news closely is considered a disaster risk reduction action. Given uncertainties about minor quakes and aftershocks during the survey period, keeping a watchful eye on disaster warnings could help stimulate an effective response. Second, preparation of emergency kits and formulation of evacuation plans are merged into a single variable because, out of all preparedness responses asked in the survey, these two actions are clear substitutes; 82.44% of households that undertook any of the two measures undertook only one action. Finally, migration intention has been shown to be a powerful predictor of actual mobility [48,49] and moving away from disaster-prone areas is one way to reduce exposure to disaster risks. Therefore, it is considered as a risk reduction measure.

It should be noted that the measurement of disaster risk reduction actions used in this paper does not strictly follow the well-developed earthquake preparation scales commonly used in the United States [27,30]. Given that the survey was carried out during the period of aftershocks, the Indian Ocean earthquake is treated as a trigger event that prompted individuals to react to potential disaster risks. In other words, risk reduction actions considered in this study are in fact responsive measures that were taken *after* the April 2012 earthquake rather than preparedness actions for future events.

The final outcome of interest is social participation. This study focuses on the role of involvement in activities through which interactions with others in the community are enhanced and exchange of information is promoted. Here social participation is derived from the question asking how often the respondent participated in community activities in the past 12 months. Individuals who participated in community activities sometimes or regularly are assigned a value of 1; those who did not are assigned a value of 0.

### Determinants of risk reduction behaviors and social participation

#### Individual characteristics

Individual characteristics that can influence risk reduction behaviors and social participation include age, gender, marital status, employment status, health, and household headship status. Experience of the 2004 tsunami is also considered. Experience is measured as the extent to which the individual was impacted by the 2004 tsunami, i.e. whether he/she experienced a loss of household members, an injury, or a property loss/damage. We focus on personal experience with disaster-induced losses since disaster response has been found to be associated with past experience [50].

#### Household characteristics

Risk reduction actions and social participation can be associated with household characteristics. These include number of household members, proportion of dependent members (those aged <5 years and those aged >60 years and over), whether the household had a disabled member, household income, and years of household settlement in the community. We also consider household location, i.e. whether the house was situated on a coastline.

It should be noted that dependent members here refer to household members who are likely to be dependent in *physical* terms. In emergency events, especially for rapid onset natural hazards like tsunami where physiology plays a key role in survivorship, very young children and older persons have a clear mortality disadvantage [51,52]. These subgroups of population are “dependent” because they rely on physically stronger household members for survivorship. We define older persons as an individual aged 60 years and over as commonly used in the Thai context [53].

#### Village characteristics

Given that risk reduction and social participation often involve collective action, characteristics of the village, namely, the number of households, percentage of female population, and percentage of women with tertiary education are also considered.

Table 1 shows the distribution of the four dependent variables and Table 2 contains summary statistics of explanatory variables. Almost two-thirds of the respondents reported having followed disaster-related news closely while about one-third mentioned that their households had prepared emergency kits and/or formed an emergency plan. One-fifth of the respondents expressed an intention to migrate from tsunami-risk areas. For a given individual, following disaster-related news closely presumably takes the least effort, followed by stockpiling emergency supplies or forming a family emergency plan, while migrating out of the area requires the most effort. The frequencies seem to reflect the effort level involved in each disaster risk reduction action. About three-fourths of the sample reported having participated in social events in a community. There are no missing observations for any of the variables included in the analysis.

**Table 1:** Distribution of dependent variables.

Disaster Preparedness Measures	Percentage
Whether closely followed the news about the earthquakes	
Yes	59.78%
No	40.22%
Whether had prepared emergency kits or formed an emergency plan	
Yes	36.80%
No	63.20%
Whether expressed desire to move after the 2012 Indian Ocean earthquake	
Yes	19.21%
No	80.79%
Whether has participated in village-based social events	
Yes	74.15%
No	25.85%
Number of observations	557

**Table 2:** Descriptive statistics of the sample.

Variables	N (%)	Mean (SD)
***Personal Characteristics***		
Head of household	336 (60.3)	
Female	306 (54.9)	
Ages 15–29	70 (12.6)	
Ages 30–39	106 (19.0)	
Ages 40–49	135 (24.2)	
Ages 50–59	122 (21.9)	
Married	391 (70.2)	
Having primary education	192 (34.5)	
Having secondary education	135 (24.2)	
Having tertiary education	51 (9.2)	
Economically inactive	113 (20.3)	
Bad subjective health	55 (9.9)	
Experienced loss of household members, injury and/or damage to property in the 2004 tsunami	255 (45.8)	
***Household Characteristics***		
Number of household members		3.865 (1.984)
Percentage of members with at least secondary education		28.659 (0.293)
Percentage of dependent members		35.337 (0.308)
Having a disabled family member	24 (4.3)	
Monthly income 10,000–19,999 THB (304–608 USD)	223 (40.0)	
Monthly income ≥ 20,000 THB (≥ 608 USD)	136 (24.4)	
Length of settlement in the area of the family relative to the respondent's age		0.563 (0.342)
Whether the household sits on a coastline	70 (12.6)	
***Village Characteristics***		
Percentage of female population		45.193 (2.499)
Percentage of female population with tertiary education		4.067 (1.535)
Number of households		623.011 (459.047)
Number of observations	557	

Notes:

1) The reference group is represented by men aged 60 years or older, who are not currently married, have no education or lower than primary education, are economically active, reported good health, did not experience loss/injury of family members in the 2004 tsunami, live in a household with no disabled family members, have a monthly income < 10,000 THB and have a house that is not located on a coastline.

2) To convert incomes from Thai Baht (THB) into US dollars (USD), the exchange rate of 32.87 THB: 1 USD was used. The exchange rate was obtained from the Bank of Thailand as of April 30, 2015: (https://www.bot.or.th/thai/statistics/financialmarkets/exchangerate/_layouts/application/exchangerate/exchangerate.aspx, retrieved on May 4, 2015)

## Empirical Model

As mentioned earlier, four binary outcomes are considered: whether or not an individual *i* followed the news about the earthquakes closely (*NEWS*_*i*_), prepared any emergency kits and/or formed any family emergency plans (*EVAC*_*i*_), expressed the desire to move from the area after the earthquakes (*MOVE*_*i*_), and participated in village-based social events.(*SOC*_*i*_) The following latent variable models are assumed:

$$NEWS_{i}^{*}=X_{i}^{\prime }\beta_{N}+\varepsilon_{Ni}$$ (1)

$$EVAC_{i}^{*}=X_{i}^{\prime }\beta_{E}+\varepsilon_{Ei}$$ (2)

$$MOVE_{i}^{*}=X_{i}^{\prime }\beta_{M}+\varepsilon_{Mi}$$ (3)

$$SOC_{i}^{*}=X_{i}^{\prime }\beta_{S}+\varepsilon_{Si}$$ (4)

The observed outcome takes the value of 1 when its associated latent variable has a positive value and 0 otherwise. In other words, where *y*_*id*_ ∈ {*NEWS*_*i*_, *EVAC*_*i*_, *MOVE*, *SOC*_*i*_}, *y*_*id*_ = 1 if $y_{id}^{*}>0$ and *y*_*id*_ = o if $y_{id}^{*}\leq 0.\;X_{i}^{'}\;$ is a matrix of all explanatory (independent) variables in the model and all regression equations share the same set of explanatory variables.*β* represents a vector of coefficients to be estimated and ε is the error term for each equation.

As explained in Fig 1, disaster risk reduction behaviors and social participation may not be independent from one another. Therefore, we employ a multivariate probit model to jointly estimate the outcomes. The multivariate probit model assumes that the four error terms are correlated according to a multivariate normal distribution such that

$$\left[\begin{array}{c} \varepsilon_{Ni}\\ \varepsilon_{Ei}\\ \varepsilon_{Mi}\\ \varepsilon_{Si} \end{array}\right]\sim N\left[\left[\begin{array}{c} 0\\ 0\\ 0\\ 0 \end{array}\right],\left[\begin{array}{ccc} 1 & \rho_{NE} & \begin{array}{cc} \rho_{NM} & \rho_{NS} \end{array}\\ \rho_{NE} & 1 & \begin{array}{cc} \rho_{EM} & \rho_{ES} \end{array}\\ \begin{array}{c} \rho_{NM}\\ \rho_{NS} \end{array} & \begin{array}{c} \rho_{EM}\\ \rho_{ES} \end{array} & \begin{array}{c} \begin{array}{cc} 1 & \;\rho_{MS} \end{array}\\ \begin{array}{cc} \rho_{MS} & \;1 \end{array} \end{array} \end{array}\right]\right]=Normal\left(0,\Omega \right)$$

where N signifies the normal distribution, 0 represents the expected value of each of the error terms and Ω is the variance-covariance matrix of the error terms. The variance-covariance matrix is notably symmetric. Each rho (ρ) represents the conditional tetrachoric correlation for each pair of outcomes, measuring the extent to which the two outcomes would covary if unobserved characteristics of an individual were indeed observed.

The cumulative probability distribution function of the above model is given by

$$\begin{array}{l} \text{Pr}\left(NEWS_{i}\;=\;1,\;EVAC_{i}\;=\;1,\;MOVE_{i}\;=\;1,\;SOC_{i}\;=\;1\right)\\ =\;\overset{\varepsilon_{Ni}}{\underset{-\infty }{\int }}\overset{\varepsilon_{Ei}}{\underset{-\infty }{\int }}\overset{\varepsilon_{Mi}}{\underset{-\infty }{\int }}\overset{\varepsilon_{Si}}{\underset{-\infty }{\int }}\phi_{4}\left(X_{i}^{\text{'}}\beta_{N},\;X_{i}^{\text{'}}\beta_{E},\;X_{i}^{\text{'}}\beta_{M},\;X_{i}^{\text{'}}\beta_{S};\;\rho_{NE},\;\rho_{NM},\;\rho_{NS},\;\rho_{EM},\rho_{ES},\rho_{MS}\right)d\varepsilon_{Ni}d\varepsilon_{Ei}d\varepsilon_{Mi}d\varepsilon_{Si}\\ ={\text{ Φ}}_{4}\left(X_{i}^{\text{'}}\beta_{N},\;X_{i}^{\text{'}}\beta_{E},\;X_{i}^{\text{'}}\beta_{M},\;X_{i}^{\text{'}}\beta_{S};\;\rho_{NE},\;\rho_{NM},\;\rho_{NS},\;\rho_{EM},\rho_{ES},\rho_{MS}\right) \end{array}$$

where *ϕ*_4_ is the joint probability density function of the fourth order. Conditional upon the empirical significance of the *ρ*’ s above, the log likelihood function becomes

$$\text{ln}L=\overset{N}{\underset{i=1}{\sum }}NEWS_{i}*\;EVAC_{i}*MOVE_{i}*SOC_{i}*\text{ln}\Phi_{4}(X_{i}^{'}\beta_{N},\;X_{i}^{'}\beta_{E},\;X_{i}^{'}\beta_{M},\;X_{i}^{'}\beta_{S};\;\rho_{NE},\;\rho_{NM},\;\rho_{NS},\;\rho_{EM},\rho_{ES},\rho_{MS}).$$

The fact that the regular maximum likelihood method would require four integrals makes the method computationally burdensome. Instead, when the number of integrals is higher than two, following Cappellari and Jenkins [54], the model is estimated using the simulated maximum likelihood (SML) method based on the Geweke-Hajivassiliou-Keane recursive simulator. The use of a multivariate probit here mirrors the conceptual framework where all outcomes of interest are jointly determined.

## Results

Tables 3 and 4 present the estimation of socio-economic determinants of social participation and three disaster risk reduction actions. Coefficient estimates from a multivariate probit model are provided in Table 3. The four binary outcomes are jointly estimated using the simulated maximum likelihood approach that is based on the seed value of 123456789 and the fact that each (simulated) error term is drawn 25 times. It should be noted that the number of draws here is larger than the recommended value of the square root of the sample size (i.e. $\sqrt{557}$) [54]. Not all pairwise *ρ*’ s are individually statistically significant. In particular, the correlation between the error term of each of the three disaster responses i.e. 1) following disaster-related news closely (*ρ* = 0.418), 2) preparing emergency kits or having an emergency plan (*ρ* = 0.135), or 3) having intention to migrate (*ρ* = 0.183), and that of social participation is statistically significant, but among the three disaster responses themselves, it is not. Nevertheless, the use of the multivariate probit model is justified by the joint significance of *ρ*’s at the 0.1% level under the likelihood ratio test.

**Table 3:** Coefficient estimates from multivariate probit regression model predicting the probability of 1) following disaster-related news closely; 2) having prepared emergency kits/plans; 3) having intention to move; and 4) having participated in community activities.

Variables	Follow the news closely	Emergency Kits/plans	Intention to move	Social participation
***Personal Characteristics***				
Head of household	-0.101	0.017	-0.228	0.055
	(0.145)	(0.150)	(0.183)	(0.168)
Female	-0.076	0.345*	0.417*	-0.057
	(0.135)	(0.141)	(0.166)	(0.152)
Ages 15–29	0.282	0.095	-0.628+	0.056
	(0.274)	(0.270)	(0.354)	(0.279)
Ages 30–39	0.229	-0.184	0.129	0.261
	(0.222)	(0.232)	(0.283)	(0.244)
Ages 40–49	0.153	-0.287	0.135	0.283
	(0.210)	(0.226)	(0.258)	(0.218)
Ages 50–59	0.173	0.067	0.0312	0.430+
	(0.205)	(0.223)	(0.264)	(0.222)
Married	0.227+	0.234	-0.042	0.018
	(0.136)	(0.143)	(0.165)	(0.151)
Having primary education	-0.319*	0.133	0.267	-0.173
	(0.153)	(0.158)	(0.179)	(0.162)
Having secondary education	-0.307	0.228	0.203	0.197
	(0.211)	(0.207)	(0.276)	(0.210)
Having tertiary education	-0.230	0.543*	0.412	-0.0068
	(0.268)	(0.276)	(0.352)	(0.276)
Economically inactive	-0.003	0.141	0.099	0.193
	(0.162)	(0.163)	(0.191)	(0.164)
Bad subjective health	0.175	0.364	0.258	-0.264
	(0.190)	(0.222)	(0.253)	(0.206)
Experienced loss of household members, injury and/or damage to property in the 2004 tsunami	0.296*	-0.017	0.393**	0.517***
	(0.123)	(0.130)	(0.151)	(0.130)
***Household Characteristics***				
Number of household members	-0.014	-0.011	0.050	-0.004
	(0.031)	(0.032)	(0.038)	(0.034)
% with ≥ secondary education	0.480+	0.169	0.258	-0.212
	(0.250)	(0.268)	(0.344)	(0.269)
% of dependent members	-0.304	0.106	0.119	0.219
	(0.243)	(0.253)	(0.295)	(0.253)
Presence of disabled member	0.446	0.675*	0.312	0.561
	(0.322)	(0.288)	(0.312)	(0.381)
Income 10,000–19,999 THB (304–608 USD)	-0.205	0.203	-0.061	-0.214
	(0.138)	(0.146)	(0.165)	(0.149)
Income ≥ 20,000 THB	-0.119	0.068	-0.348	-0.070
(≥ 608 USD)	(0.164)	(0.172)	(0.219)	(0.179)
Length of settlement	0.128	-0.126	0.109	0.002
	(0.188)	(0.190)	(0.246)	(0.204)
House on a coastline	-0.084	0.195	0.716***	-0.114
	(0.177)	(0.173)	(0.193)	(0.184)
***Village Characteristics***				
% female population	-0.106***	-0.165***	-0.183***	-0.058+
	(0.032)	(0.032)	(0.047)	(0.032)
% females with tertiary education	0.167***	0.216***	0.369***	0.115**
	(0.039)	(0.042)	(0.056)	(0.041)
Number of households	-0.0006***	0.0002	-0.0004**	-0.0001
	(0.0002)	(0.0001)	(0.0002)	(0.0002)
Constant	4.603**	5.410***	5.025*	2.537
	(1.514)	(1.481)	(2.117)	(1.553)
***ρ*** (Evacution & News)	0.094			
	(0.075)			
***ρ*** (Emergency & Move intention)	0.130			
	(0.091)			
***ρ*** (News & Move intention)	0.061			
	(0.088)			
***ρ*** (News & Social participation)	0.418***			
	(0.079)			
***ρ*** (Emergency & Social participation)	0.135+			
	(0.081)			
***ρ*** (Move intention & social participation)	0.183+			
	(0.103)			
**LR joint test of *ρ*’s**	33.258***			
**Wald test: Overall significance**	87.810***			
**Log psuedolikelihood**	-1125.59			
**Observations**	557			

Notes:

Coefficient estimates are calculated based on a simulated maximum likelihood approach. The Geweke-Hajivassiliou-Keane (GHK) recursive simulator is used; each of the four error terms is simulated and drawn 25 times based on the seed value of 123456789.

Robust standard errors are in parentheses.

*** p<0.001,

** p<0.01,

* p<0.05,

^+^ p<0.1.

**Table 4:** Marginal effects from multivariate probit model.

Variables	Follow the news closely	Emergency Kits/plans	Intention to move	Social participation
***Personal Characteristics***				
Head of household	-0.035	0.005	-0.047	0.016
	(0.051)	(0.048)	(0.037)	(0.050)
Female	-0.026	0.109***	0.086**	-0.017
	(0.047)	(0.044)	(0.034)	(0.045)
Ages 15–29	0.098	0.030	-0.129+	0.017
	(0.095)	(0.086)	(0.072)	(0.082)
Ages 30–39	0.080	-0.058	0.026	0.077
	(0.077)	(0.074)	(0.058)	(0.072)
Ages 40–49	0.053	-0.091	0.028	0.084
	(0.073)	(0.071)	(0.053)	(0.064)
Ages 50–59	0.060	0.021	0.006	0.127**
	(0.071)	(0.071)	(0.054)	(0.065)
Married	0.079+	0.074+	-0.009	0.005
	(0.047)	(0.045)	(0.034)	(0.045)
Primary education	-0.111**	0.042	0.055	-0.051
	(0.053)	(0.050)	(0.036)	(0.048)
Secondary education	-0.107	0.072	0.042	0.058
	(0.073)	(0.065)	(0.056)	(0.062)
Tertiary education	-0.080	0.172*	0.084	-0.002
	(0.093)	(0.087)	(0.072)	(0.081)
Economically inactive	-0.001	0.045	0.020	0.057
	(0.056)	(0.052)	(0.039)	(0.048)
Bad subjective health	0.061	0.115+	0.053	-0.078
	(0.066)	(0.070)	(0.052)	(0.061)
Experienced loss of household members, injury and/or damage to property in the 2004 tsunami	0.103**	-0.005	0.081***	0.153***
	(0.042)	(0.041)	(0.030)	(0.037)
***Household Characteristics***				
Household members	-0.005	-0.004	0.010	-0.001
	(0.011)	(0.010)	(0.008)	(0.010)
% with ≥ secondary education	0.167*	0.054	0.053	-0.063
	(0.087)	(0.085)	(0.071)	(0.079)
% of dependent members	-0.106	0.034	0.024	0.065
	(0.084)	(0.080)	(0.061)	(0.075)
Presence of disabled member	0.156	0.214*	0.064	0.166
	(0.112)	(0.090)	(0.064)	(0.112)
Income 10,000–19,999 THB (304–608 USD)	-0.071	0.064	-0.012	-0.063
	(0.048)	(0.046)	(0.034)	(0.044)
Income ≥ 20,000 THB (≥ 608 USD)	-0.041	0.021	-0.071	-0.021
	(0.057)	(0.054)	(0.044)	(0.053)
Length of settlement	0.045	-0.040	0.022	0.001
	(0.065)	(0.060)	(0.050)	(0.060)
House on a coastline	-0.029	0.062	0.147***	-0.034
	(0.062)	(0.055)	(0.038)	(0.054)
***Village Characteristics***				
% female population	-0.037***	-0.052***	-0.038***	-0.017*
	(0.011)	(0.009)	(0.009)	(0.009)
% females with tertiary education	0.058***	0.068***	0.076***	0.034***
	(0.013)	(0.012)	(0.010)	(0.012)
Number of households	-0.0002***	0.00007	-0.0001***	-0.00004
	(0.00005)	(0.00005)	(0.00003)	(0.00005)

Notes: Standard errors are in parentheses and are calculated using the delta method.

*** p<0.001,

** p<0.01,

* p<0.05,

^+^ p<0.1

To be able to interpret the results in terms of the probability, for each equation, marginal effects of all explanatory variables are given in Table 4. Marginal effects show the change in probability when the predictor increases by one unit. At the individual level, being female is associated with 10.9% and 8.6% higher probabilities of having emergency kits and/or an emergency plan, and having an intention to migrate. Individuals aged 50–59 years are more likely to participate in social events compared to those aged 60 years and over. Being married is linked with a 7.9% and a 7.4% increase in the probabilities of following disaster-related news closely and forming an emergency plan respectively. Respondents with tertiary education and those having a disabled person in the household have a greater probability to prepare for emergency kits and/or establishing a family emergency plan. Unsurprisingly, individuals living in house located on a coast are 14.7% more likely to express a desire to move away from tsunami-risk areas. The most important predictor is, as hypothesized, whether the individual was affected by the 2004 tsunami in terms of loss of property or household members; this characteristic is a key driver of the likelihood of following the news closely (10.3%), intention to migrate (8.1%), and social participation (15.3%).

Some village characteristics are also statistically associated with the four outcomes. In general, an increase in the proportion of women in the village leads to a reduction in both disaster responses and social participation. However, the opposite is true with respect to the proportion of women with tertiary education. The greater the proportion of women with tertiary education in the village, the greater the likelihood that the individual would follow disaster-related news closely, prepare emergency kits and/or initiate a family emergency plan, and intend to migrate as well as participate in village-based activities.

In order to determine the pathway in which social participation affects different types of disaster responses, drawing on the predicted joint probabilities explained earlier, conditional probabilities are provided in Table 5. The first three rows show conditional probabilities of undertaking each disaster risk reduction measure estimated with bivariate probit models using the same vector of independent variables as in the multivariate probit model. They are provided in order to show the relationship between social participation and a given disaster response more clearly, and to illustrate the impact of social participation on one disaster response *irrespective* of the others. The last two rows display conditional probabilities estimated with the multivariate probit model containing possible joint events of carrying out three disaster risk reduction behaviors altogether. The first two columns illustrate the probabilities of carrying out disaster risk reduction measures conditional on having some social participation and having no social participation respectively. The subsequent column shows the paired difference between the two conditional probabilities and the final column shows results of the t-test performed on the paired difference.

**Table 5:** Conditional probabilities of risk reduction behaviors.

Events	Conditional on *SOC* ^c^ = 1^a^	Conditional on *SOC* = 0^a^	Paired Difference^b^	T-Test Statistics
*NEWS* ^c^ = 1	0.660	0.401	0.259	195.119***
	(0.151)	(0.153)	(0.001)	
*EVAC* ^c^ = 1	0.388	0.308	0.079	72.043***
	(0.207)	(0.191)	(0.001)	
*MOVE* ^c^ = 1	0.204	0.139	0.065	31.475***
	(0.197)	(0.155)	(0.002)	
*NEWS* = 0, *EVAC* = 0, *MOVE* = 0	0.203	0.386	-0.183	-61.235***
	(0.152)	(0.204)	(0.003)	
*NEWS* = 1, *EVAC* = 1, *MOVE* = 1	0.085	0.033	0.052	22.398***
	(0.099)	(0.047)	(0.002)	

Notes:

^a^ Standard deviations are given in parentheses.

^b^ Standard errors are provided in parentheses and they are equal to standard deviations divided by the square root of the number of observations (557).

^c^ *SOC* refers to “social participation”; *NEWS* refers to “following disaster-related news closely”; *EVAC* refers to “preparation of emergency kits or having family emergency plan”; and *MOVE* refers to “intention to migrate”.

*** p<0.001,

** p<0.01,

* p<0.05,

^+^ p<0.1

It can be seen from Table 5 that with the absence of social participation, the probabilities of undertaking each of the preparatory measures, i.e. following disaster-related news closely, preparing emergency kits and/or having a family emergency plan, and intending to migrate are 40%, 31% and 14% respectively. Yet, conditional on social participation, the likelihood of each event increases to 66%, 39% and 20% respectively. Likewise, the probability of undertaking all risk reduction measures is higher by 5.2% in the presence of social participation (*NEWS* = 1, *EVAC* = 1, *MOVE* = 1|*SOC* = 1). While the probability of not undertaking any of the risk reduction measures is almost 40% given no social participation (*NEWS* = 0, *EVAC* = 0, *MOVE* = 0|*SOC* = 0), it reduces to only 20% conditional on social participation (*NEWS* = 0, *EVAC* = 0, *MOVE* = 0|*SOC* = 1).

## Discussion

This paper examines the determinants of and the relationships between disaster risk reduction behaviors and social participation using the case study of disaster response during the Indian Ocean earthquakes in 2012 in Phang Nga province, Thailand. In particular, we investigate three disaster risk reduction behaviors (namely, following disaster-related news closely, having emergency kits and/or a family emergency plan, and having an intention to migrate), model the probability to participate in community activities, and quantify the relationship between disaster risk reduction behaviors and social participation through the estimation of conditional probabilities.

Our main finding is that the likelihood of undertaking risk reduction actions is highly correlated with social participation. The probabilities of following disaster-related news closely, preparing for emergency supplies or having a family emergency plan, and having an intention to migrate significantly increases for individuals who have engaged in community activities. While recent studies have shown that social capital increases disaster preparedness [23,42], the dimensions of social capital considered are related to trust and social cohesion. We argue that social participation is an important dimension of social capital particularly in the case of risk reduction actions because it broadens one’s social connections, facilitates exchanges of information and increases encouragement/peer pressure. As evident in previous literature, social participation brings about positive externality such as increasing leisure-time physical activity [55], smoking cessation [56] and survival in old age [57]. This suggests that promoting civic and social engagement can also be beneficial to disaster mitigation.

Furthermore, we also explored relevant individual-, household- and community-level variables associated with disaster risk reduction behaviors. It is found that being badly affected by the 2004 Indian Ocean tsunami is a key driver of preventive actions, especially following disaster-related news closely and having an intention to migrate. However, tsunami experience is not significantly associated with the likelihood of preparing emergency kits or having a family emergency plan. Consistent with previous studies, while prior disaster experience is positively correlated with increased general preparedness [27,58], not all types of preparedness actions naturally increase with experience [59].

Indeed, some disaster preparedness tasks are easier to implement than others. Closely following the news about the earthquakes only requires an individual to turn on their television set or update the situation with their neighbors, whereas assembling an emergency kit and having a family emergency plan require stockpiling of necessary supplies and coordination among family members respectively. The latter entails more efforts and strategic planning. This is consistent with the fact that we observe that individuals with tertiary education are more likely to gather supplies and/or implement a family plan while prior disaster experience does not contribute to such action.

Likewise, while previous empirical studies from the United States report mixed evidence regarding disability status and disaster preparedness [60–62], we find that the presence of household members with a disability increases the likelihood of having disaster supplies or an emergency plan. Preparedness items can mitigate adverse impacts especially for persons with disability who are most vulnerable during the time of disasters. Our finding underlines the importance of promoting preparedness among vulnerable groups.

Risk reduction behaviors also vary considerably by gender. It is found that women are both more likely to stockpile supplies or form a family emergency plan and have a higher intention to move away from tsunami-risk zones. One explanation is that women perceive disaster events or threats as more serious and hazardous compared to men [63,64] and this consequently translates into greater risk reduction actions. Nevertheless, at the community-level, we find that the probabilities of undertaking preparedness measures and intention to migrate increase substantially only in a community with a greater proportion of women with tertiary education. Living in a community with a large proportion of highly educated women likely promotes personal disaster preparedness because education increases access to disaster-related information and socioeconomic resources. Since women are more likely to have denser social ties comprising a higher proportion of kin and neighbors than men [65], having highly educated women in a community could result in a spillover effect on risk reduction behaviors.

The positive community-level effects of women's education on social participation and risk reduction actions are noteworthy. Recent evidence points to the similarly vital role of female education in reducing vulnerability: from minimizing malaria risk in children [66], lowering disaster-related mortality [67], to enhancing adaptive capacity [68]. This suggests that investing in and broadening girls’ access to quality education can have far-reaching benefits especially in times of a changing climate and rising frequency and severity of natural disasters.

Jointly estimating outcomes of interest, we are able to account for the interdependence of the decision to undertake disaster preparedness measures, intention to migrate and social participation. However, since this study is based on cross-sectional data, we have to rely on the assumption that individuals make decisions on these actions *simultaneously*. A different timing assumption is plausible. Given that the survey was collected after the 2012 earthquakes, it is not unreasonable to think that disaster risk reduction measures were employed after the incident while engagement in community-based activities had previously been pursued. In this case, social participation should be modeled as an endogenous independent variable that explains disaster preparedness outcomes. Such modeling technique is principally more appropriate with panel data, nevertheless.

## Conclusion

Without doubt, preparing for a natural disaster is an efficient way to minimize its adverse impact. It is therefore important to understand not only factors that may hinder risk reduction behaviors but also the ones that promote them. While it is not possible or difficult to alter demographic characteristics associated with disaster risk reduction actions such as age and gender, certain social characteristics can be improved. Our finding that engagement in community-based activities increases disaster preparedness and intention to move away from disaster-risk areas suggests that promoting social participation may generate a positive externality in reducing vulnerability and disaster risk.

## Supporting Information

S1 File Participant Information Sheet. (PDF)
