# Supplementary material for: Social Participation and Disaster Risk Reduction Behaviors in Tsunami Prone Areas
Source: PLoS One. 2015 Jul 8;10(7):e0130862. doi: 10.1371/journal.pone.0130862 (PMC4495990; doi:10.1371/journal.pone.0130862)
Supplement: S1 File — (PDF) [file pone.0130862.s001.pdf]

Participant Information Sheet

1. **Project title** Understanding Social Barriers to Coping with and Adapting to Extreme Climate Events

My name is _________________. I am a trained interview staff working for the College of Population Studies, Chulalongkorn University in Bangkok. We are working on a project looking at household responses to the Indian Ocean Earthquake on 11 April 2012 and the aftershocks that followed the earthquake event. I would like to invite you to take part in this research project. Before you decide you need to understand why the research is being done and what it would involve for you. Please take time to read the following information carefully. Please ask questions if anything you read is not clear or would like more information. Please take your time to decide whether or not to take part.

2. **Principal investigators**

Dr. Vipan Prachuabmoh
Dr. Raya Muttarak
Dr. Wiraporn Pothisiri
Office: College of Populations Studies, Chulalongkorn University, Bangkok 10330
Tel: 02-218-7340, 02-218-7342

3. **Research objectives**
   1) To fulfil the main goal of Chulalongkorn University as the "pillar of the Kingdom" by applying the results from the project to enhance adaptive capacity at the individual and community level.
   2) To collect information on demographic differential vulnerability to impacts of natural disasters, recovery and responses.
   3) To identify which population subgroups are vulnerable in order to draw a policy implication on adaptation and responses to climate change which are suitable to different locations and subgroups of populations.
4. **Sample population**

   One adult member per household living in tsunami-risk areas in Phang Nga. 500 households will be interviewed.
5. **Data collection procedure**

   You will be interviewed based on a structured questionnaire by trained interview staff. The interview typically takes 15 minutes.
6. **Risks**

   There are no known risks for you in this study.
7. **Benefits**

   This research project will not have a direct benefit to the participants but the project is expected to contribute to the understanding of demographic differential vulnerability to impacts of natural disasters, recovery and responses. This knowledge will be used identify which population subgroups are vulnerable in order to draw a policy implication on adaptation and responses to climate change which are appropriate to areas and population subgroups.

8. **Expenses and payments**

   You will not receive any compensation upon participating in this project.

9. **Choice of participation**

   Taking part in the research is entirely voluntary.

10. **Confidentiality**

    All information which is collected about you during the course of the research will be kept strictly confidential and only the named principal investigators can access the raw data. Your name and address will be removed so that you cannot be recognized.

11. **Right of participants**

    You may decide to stop being a part of the research study at any time without explanation. You have the right to ask that any data you have supplied to that point be withdrawn/destroyed. You have the right to omit or refuse to answer or respond to any question that is asked of you. You have the right to have your questions about the procedures answered.

12. **For further information**

    Dr. Wiraporn Pothisiri will be glad to answer your questions about this study at tany time. You may contact her at Tel: 02-218-7340, 02-218-7342.
